# Editorial: Nutrition and Metabolism in Rheumatic Diseases

**DOI:** 10.3389/fmed.2019.00101

**Published:** 2019-05-08

**Authors:** Helena Canhao, Kayo Masuko, Hiroshi Nakamura

**Affiliations:** ^1^CEDOC, Epidoc Unit, NOVA Medical School, Universidade Nova de Lisboa, Lisbon, Portugal; ^2^Rheumatology Department, CHULC-Hospital Curry Cabral, Lisbon, Portugal; ^3^International University of Health and Welfare, Clinical Research Center, Tokyo, Japan; ^4^Sanno Medical Center, Tokyo, Japan; ^5^Sanno Hospital, Tokyo, Japan

**Keywords:** nutrition–clinical, metabolism, rheumatic and musculoskeletal diseases (rmds), nutrition, microbiome

Every day, we eat, digest and absorb nutrients through our gastrointestinal mucosa into circulation resulting in a cascade of biochemical responses within cells to finally produce energy. On the other hand, skewed or insufficient nutritional intake, decreased physical activity and/or dysregulated metabolic responses may lead to a perpetuation, deterioration or even occurrence of inflammation or chronic diseases. Recent accumulation of investigations points to an essential role of nutrition and metabolic regulations in the pathogenesis and pathophysiology of many diseases, including rheumatic disorders. In particular, the concept of “immunometabolism” has been recently introduced showing how metabolic pathways would modulate functions of immune cells. Further, a potential contribution of adipocytes, or of visceral fat in systemic or local inflammation and rheumatic arthropathies has been actively discussed. This Research Topic, aimed to focus on the potential role of nutrition and metabolism in rheumatic diseases. It consists of a set of five papers—two mini-reviews, one research original paper, and finally a case report followed by a hypothesis theory paper.

Guerreiro et al. present a mini review “Diet, Microbiota, and Gut Permeability—The Unknown Triad in Rheumatoid Arthritis” acknowledge and review the relationship between rheumatoid arthritis and distinct gut microbiota composition in comparison to healthy individuals. In the end they elicit critical questions that remain to be elucidated: Are the clinical benefits associated with the selective growth of a “healthy gut microbiota.” What is the relevance of intestinal permeability in this equation since both diet and microbiota seem to change its functionality? How the microbiome fluctuates in relation to diet, and how disease activity may be influenced by changes in diet, microbiota or diet-intestinal microbiota equilibrium. These questions show that large room for research exist in this field.

Masuko reviewed “A Potential Benefit of “Balanced Diet” for Rheumatoid Arthritis.” In this paper Masuko discussed that rheumatoid arthritis patients should be advised that a balanced diet that includes appropriate amounts of carbohydrate, especially dietary fiber, is important for maintaining the symbiosis of intestinal flora, which could be beneficial for preventing autoimmunity.

In an original paper “Chemerin and PEDF Are Metaflammation-Related Biomarkers of Disease Activity and Obesity in Rheumatoid Arthritis” Tolusso et al. aimed to determine whether PEDF and Chemerin are biomarkers of inflammation related to fat accumulation in RA and to investigate whether weight loss associates with clinical disease improvement through the modification of fat-related biomarkers in overweight/obese RA with low-moderate disease. They concluded that PEDF and Chemerin arose as biomarkers of obesity and metaflammation respectively, providing a link between chronic inflammation and excess of body weight in RA. Therefore, BMI reduction of at least 5% in obese RA allowed better disease control without modifying RA treatment.

Finally, the last two papers from Lattanzio and Imbesi focused on the relationship between fibromyalgia symptomatic control and diet strategy. The first one, “Fibromyalgia Syndrome: A Case Report on Controlled Remission of Symptoms by a Dietary Strategy” reported a case of symptoms' improvement in a fibromyalgia patient after a diet allowing a proper absorption of tryptophan assumed with food, while avoiding, or at least minimizing the presence of interfering non-absorbed molecules, such as fructose and sorbitol. The authors suggest the implementation of a clinical trial to validate this strategy in a large scale. In the last paper Lattanzio develop a hypothesis and theory paper “Fibromyalgia Syndrome: A Metabolic Approach Grounded in Biochemistry for the Remission of Symptoms,” where she developed the theory of the relationship between a diet with low tryptophan absorption and fibromyalgia symptoms.

This set of papers show how this field is in an emergent phase and how much research is needed in this area of knowledge.

## Author Contributions

All authors listed have made a substantial, direct and intellectual contribution to the work, and approved it for publication.

### Conflict of Interest Statement

The authors declare that the research was conducted in the absence of any commercial or financial relationships that could be construed as a potential conflict of interest.

